# A Comprehensive Predictive-Learning Framework for Optimal Scheduling and Control of Smart Home Appliances Based on User and Appliance Classification

**DOI:** 10.3390/s23010127

**Published:** 2022-12-23

**Authors:** Wafa Shafqat, Kyu-Tae Lee, Do-Hyeun Kim

**Affiliations:** 1 Division of Information and Communication Engineering, Kongju National University, Cheonan 331717, Republic of Korea; 2 Computer Engineering Department, Ara Campus, Jeju National University, Jeju-si 63243, Republic of Korea

**Keywords:** energy consumption, energy cost, optimal scheduling, smart homes, user classification, appliance classification, grey wolf optimization, particle swarm optimization

## Abstract

Energy consumption is increasing daily, and with that comes a continuous increase in energy costs. Predicting future energy consumption and building an effective energy management system for smart homes has become essential for many industrialists to solve the problem of energy wastage. Machine learning has shown significant outcomes in the field of energy management systems. This paper presents a comprehensive predictive-learning based framework for smart home energy management systems. We propose five modules: classification, prediction, optimization, scheduling, and controllers. In the classification module, we classify the category of users and appliances by using k-means clustering and support vector machine based classification. We predict the future energy consumption and energy cost for each user category using long-term memory in the prediction module. We define objective functions for optimization and use grey wolf optimization and particle swarm optimization for scheduling appliances. For each case, we give priority to user preferences and indoor and outdoor environmental conditions. We define control rules to control the usage of appliances according to the schedule while prioritizing user preferences and minimizing energy consumption and cost. We perform experiments to evaluate the performance of our proposed methodology, and the results show that our proposed approach significantly reduces energy cost while providing an optimized solution for energy consumption that prioritizes user preferences and considers both indoor and outdoor environmental factors.

## 1. Introduction

With the advancements of technology energy management have become a global concern as energy is amongst the most imperative facets of technological development. In smart homes, efficient energy management is grabbing more attention day by day because of the growing number of smart devices, which leads to more consumption of energy and results in heavy electricity bills. It is challenging to manage energy consumption to reduce the cost by not causing discomfort to the residents. A smart home consists of different smart appliances, different sensors, actuators, and users. The end goal for an energy management system of a smart home is to minimize energy cost and utilization and at the very same time maximize user comfort. To achieve this optimal solution, primary components and tasks need to be considered simultaneously that include identification of user behaviors, and appliance types, monitoring the interaction of users with the appliances, predicting future patterns of usage, interaction, and cost, finding optimal schedules to run the appliances by not compromising on user comfort, and generating control rules to manage and control smart home devices. Lately, there have been lots of studies and experiments performed in this area focusing on the optimization, scheduling, control, and effective classification of users. However, many studies focus on limited aspects at a time. Therefore, the first contribution of this study is to provide a complete framework for the smart home energy optimization problem i.e., starting from the effective classification of appliances and users, prediction of different energy consumption patterns, user behavior patterns, and future energy consumption patterns, optimization of desired parameters and based on optimal parameters, and appliance consumption patterns, generating optimal schedules for these appliances and finally generating control rules through the controller to regulate these smart home appliances. The second contribution of this study is to build a customized smart home controller for optimal energy management. Energy consumption patterns are based on user behaviors; thus, different household users can have different behaviors and can present different patterns of energy consumption. Similarly, different appliances have different energy powers and different usage patterns. Therefore, there is a need for a customized energy management system that takes care of both the user behaviors, appliance categories, and the nature of interaction among smart home users and smart appliances. Contrary to other studies, besides the standard categories of appliances, we take a step ahead toward contextual understanding by estimating the effect of each user class’s interaction with all types of home appliances. The third contribution is to use a hybrid of particle swarm optimization (PSO) and grey wolf optimization (GWO) algorithms to formulate multi-objective maximization and minimization problems for optimal scheduling of the appliances.

In the field of nonlinear sciences, experts have studied different optimization techniques for various applications. In [1], authors have focused on the qualitative data present in the financial reports instead of the quantitative data. Authors use Harris Hawks Optimization (HHO) for feature selection and a Deep Neural Network-based Deer Hunting Optimization (DNN-DHO) to detect fraud reports in financial statements. In addition, Luo et al. [2] introduced a Reference Signal Optimization Algorithm (RSOA) to enhance the indoor Bluetooth positioning. This paper uses supervised learning and performs optimization of environmental factors to improve the accuracy of indoor positioning. In [3], scholars have proposed an enhanced real-time single-sensor appliance classification and monitoring system leveraging convolutional neural networks and transfer learning. The proposed model helps to identify defective behavior, failing appliances, and automatic load control. In another study [4], device states are inferred by using electric signals from smart home appliances along with temperature and humidity sensors input. A variation of the Probabilistic K-Nearest Neighbor (PKNN) approach is used to classify the appliances. This proposed approach helps in reducing computational time as compared to the traditional PKNN approach. Other machine learning approaches have been used to classify appliances and their abnormal consumption patterns such as long short-term memory (LSTM), Naïve Bayes, and Decision trees, etc. [5,6,7]. Besides the classification of appliances, the psychological aspects of smart home users are also of key importance. As users have different lifestyles depending upon their earnings, age, and routine, therefore, they have different patterns of energy consumption. Numerous studies have focused on user classification and activity recognition to identify different consumption patterns and then proposed different techniques based on user categories to minimize and control smart home energy. In [8], a multiagent system is being proposed to monitor the smart home user for activity classification. User location, sequence of his activities, and other temporal information are utilized for the prediction of user behavior in smart homes. Other techniques such as Neural Networks (NN) [9,10,11,12], fuzzy logic [13], and statistical methods [14] are also used for user activity recognition. Ref. [9] proposed an adaptive control of home environments (ACHE) that observes the lifestyle, actions, and desires of users to accommodate their needs. ACHE has two objectives: to understand, the user needs to minimize manual adjustments and the second objective is to conserve energy. The authors use neural networks to build their architecture. Ref. [10] introduces a methodology to cluster daily user activity analysis within smart homes. Their work is based on a self-adaptive neural network which is named as growing self-organization maps. Refs. [11,12] introduce a person-tracking system for EasyLiving project in smart environments. The system can track multiple people and multiple activities. To reduce electricity consumption and electricity bill prices, it is vital that the appliances are not scheduled at peak hours. It is equally important to find out the optimal time and duration of an appliance to be used in a day so that energy consumption and cost both can be reduced. A single objective function-based optimization problem is introduced in [15] to help reduce the total energy consumption cost. Ref. [16] is a similar study that introduces an objective function for energy optimization. The goal of the objective function is to minimize energy costs and maximize user preferences and user comfort. PSO has recently gained much importance and attention from the research community in solving optimization problems in smart homes. In [17], PSO is used for the task scheduling of appliances based on user preferences. Here, the charging and discharging schedules for electric vehicles are generated on an hourly basis, and schedule and control rules for heaters to turn them off or on are also generated on hourly bases. In our previously proposed studies, we have also utilized PSO-based optimization techniques and formulated objective functions to solve different maximization and minimization objectives in smart homes [18,19,20]. In [21], a Genetic Algorithm (GA) based model for optimized scheduling of smart home appliances is proposed that focuses on reducing the electricity cost and load peak value. Other techniques, such as linear integer programming [22], GWO [23,24,25], wind-driven optimization [26], and mixed integer linear programming (MILP) [27], etc., have also been widely utilized to solve optimal scheduling problems in smart homes. Smart home controllers operate actuators and appliances. A simple controller generates control commands to turn on an appliance or to turn it off. To minimize energy consumption cost, effective definitions of control commands are important to control smart home devices. In [28], the designed control rules automatically control the appliances to minimize the energy consumption cost by prioritizing the user preferences simultaneously.

As discussed above, many studies and techniques have been presented in the literature to minimize the energy consumption cost in smart homes by either providing optimal controls, effective classification of users or appliances, and optimal scheduling of smart home appliances, etc. However, to the best of our knowledge, none of the studies have simultaneously focused on all these components. Therefore, in this paper, we propose a comprehensive predictive-learning framework for optimal scheduling and control of smart home appliances. Our main contributions are:

- We propose a complete five-layered architecture for energy management in smart homes;
- Our model classifies users and appliances and considers the interaction level between them for optimal scheduling and control of smart home appliances;
- We propose objective functions to find an optimal schedule for all appliances with a maximum user preference value and minimum energy consumption and cost;
- We propose a novel algorithm based on the combination of Grey Wolf optimization (GWO) and particle swarm optimization (PSO) for optimized scheduling.

The next section presents the complete framework, conceptual design, and algorithmic details of the proposed study. Section 2 introduces the proposed methodology, which is subdivided into Section 2.1: conceptual model, Section 2.2: architectural overview, and Section 2.3: algorithmic approaches. Next, Section 3 demonstrates the performance evaluation, and we conclude our research work in Section 4.

## 2. Proposed Methodology

Prediction of energy consumption in the housing sector is a crucial and challenging task as it involves and varies according to user behavior. In this section, we discuss the implementation of machine learning-based situation recognition, optimization, and control technology to find an optimal schedule that considers maximum user preference value and entails minimum energy consumption and cost for all the appliances. We will first discuss the conceptual model of the proposed approach and would proceed with the architectural view and the algorithmic approaches considered in the proposed methodology.

### 2.1. Conceptual Model

**Figure 1:**
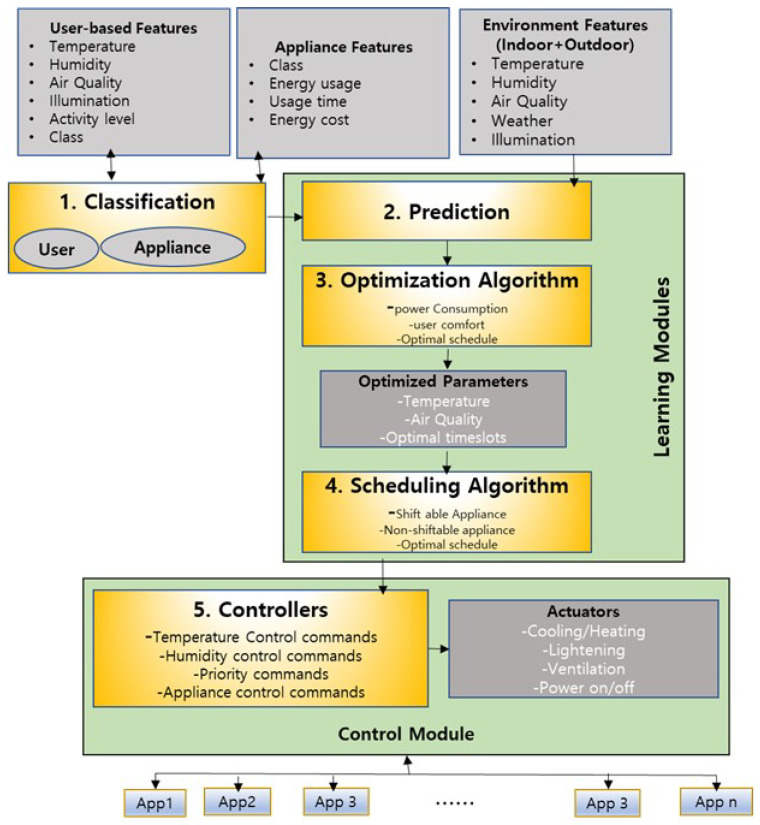
Conceptual view of the proposed methodology.

In this work, we mainly focus on user-based features, appliance features, and environmental features of both indoor and outdoor environments. The user-based features include the start and end time of an activity, duration of the activity, location, type of appliance, and information about the place used in the household during the start and end time. Appliance features comprise the name and ID of the appliance, start and end time of appliance usage, duration, energy consumption, and energy cost. We consider temperature, humidity, air quality, and the illumination of the outdoor and indoor environment. The conceptual view of the proposed methodology is shown in Figure 1, which contains five primary modules:

1. Classification;
2. Prediction;
3. Optimization;
4. Scheduling;
5. Control.

As can be seen in Figure 1, the classification module is responsible for user and appliance classification. We categorize the users’ characteristics into five classes depending on the user activity, total hours of motion, and appliance usage details. We classify the user as a super-active user, semi-active user, less-active user, rarely-active user, or not-active user. For appliance classification, we consider two factors: consumption of energy by the appliances and the type of appliance, i.e., shiftable appliances, non-shiftable appliances, and controllable appliances. Based on the user and appliance classification and the indoor and outdoor environment features, we implement the LSTM based prediction algorithm to forecast an appliance’s energy consumption and cost on an hourly basis. In the optimization and scheduling module, our goal is to find an optimal schedule for all appliances which would generate minimum energy cost while giving the highest priority to user preferences. For optimization, we focus on energy cost-based optimization and appliance category-based optimization, whereas for scheduling, we consider user comfort, appliance category, and their related energy cost according to season and time period e.g., peak hours off-peak hours, mid-peak hours, etc. In the control module, we define the control rules based on the historical usage pattern, current user activities, schedules, environmental factors, and the predicted energy profile.

### 2.2. Architectural Overview

**Figure 2:**
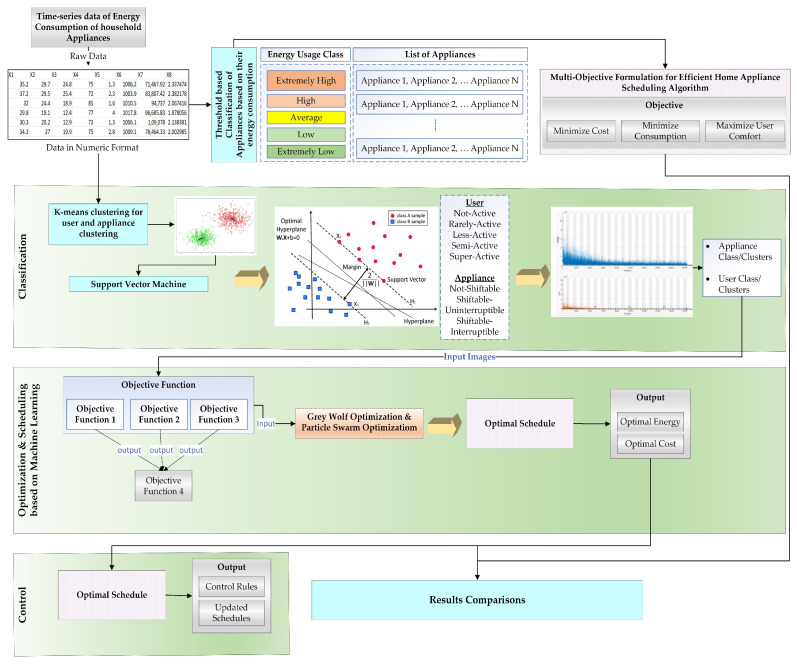
Architectural overview of the proposed methodology.

The architectural overview of the proposed methodology is presented in Figure 2. The input to our proposed model is time-series energy consumption data of household appliances and user activity data along with indoor and outdoor environmental condition data. Appliances have been classified depending on their energy consumption. We have defined the energy usage class of appliances in five categories, i.e., extremely high energy usage, high energy usage, average energy usage, low energy usage, and extremely low energy usage. The user-based feature data consist of activity as well as the motion information of the user. We have collected user, appliance, and energy consumption data from two data sources. The user activity data have been obtained from [29], which categorizes activity into showering, sleeping, breakfast, leaving, toileting, dinner, drinking, idle/unlabeled, lunch, snack, spare time/TV, computer, laundry, and grooming. The sensor data in the user activity dataset have been divided into time intervals of 60 s length. The data have been collected over a period of 21 days with a total of 17 sensors. Currently, there is no availability of open-source data that considers all the features we aim to use in our proposed work. Therefore, we have synthetically prepared the dataset according to our requirements. According to the sensor location, type, place, usage time, and activity data in [29], we assigned the appliances and synthetic duration to the activities of the user. We referred to [30] for relating energy consumption values and energy cost calculation. We have obtained the indoor environment data through indoor sensors and outdoor environment data have been collected from [31]. Table 1, Table 2, Table 3 and Table 4 present the features of user activity data, sensor data, appliance, and outdoor data that we have considered as input to our proposed methodology.

The user, appliance, and indoor and outdoor data are fed as input to the classification module. We have defined user categories based on their activity level and appliance category through their level of energy consumption. We use K-means clustering and a support vector machine algorithm to categorize and classify appliances and new users. In the prediction module, we use the LSTM based algorithm to predict a user category and the future energy consumption and energy cost depending on the user category and preferences. In the optimization and scheduling phase, our main goal is to define the objective to minimize energy consumption and energy cost and maximize the priorities given to user preferences. For scheduling, we use grey wolf optimization (GWO) and particle swarm optimization (PSO) algorithms for optimized scheduling of appliances according to indoor and outdoor environmental factors, energy consumption, and cost and user preferences. Control rules have been used in the control module to control the usage of appliances according to the schedule while prioritizing user preferences and minimum energy consumption and cost.

### 2.3. Algorithmic Approaches

In the section, we define the functionality of each proposed module and the algorithmic approaches we have used for each module. We formulate objective functions for optimized scheduling and controlling of smart home appliances. We will describe in detail the classification, prediction, and scheduling algorithms we have used, the input and output data of each module, and the processing steps.

#### 2.3.1. Classification

**Figure 3:**
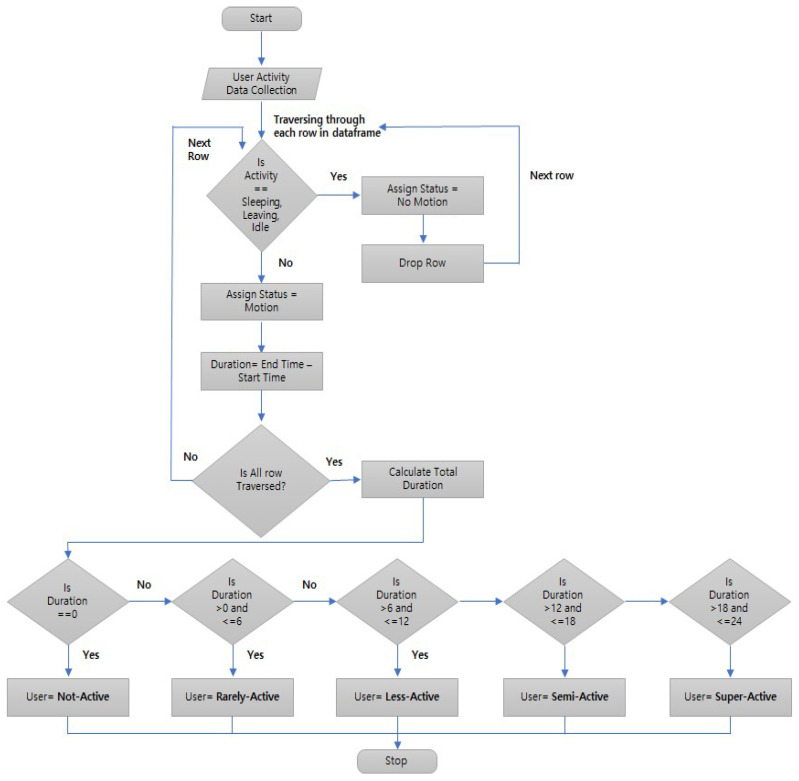
Flowchart for defining user classes.

In the classification module, we focus on the categorization and classification of users and appliances. Based on the activity and motion detail, we have defined five categories for users i.e., super-active, semi-active, less-active, rarely-active, and not-active. First, we collect the user activity data from [29] and check whether the activity is referring to sleeping, leaving, or idle. For these activities, we assign the status “No Motion”. For the other activities detected by the motion sensors, we assign the status “Motion”. We calculate the total duration of motion for each user. If the duration is zero hours, then the user is considered as not-active. If the duration is greater than zero hours and less than or equal to six hours, the user is classified as rarely-active. User with motion duration between six and twelve hours is classified as less-active. If the duration is greater than twelve hours but less than or equal to eighteen hours, the user is classified as semi-active. If the duration is greater than eighteen hours, the user is classified as super-active. The flowchart for defining user classes is shown in Figure 3.

For appliances, we have considered predefined classes such as non-shiftable, shiftable-uninterruptible, and shiftable-interruptible appliances based on [32]. Non-shiftable appliances are those appliances that cannot be interrupted and should remain switched ON continuously during the scheduled period. Examples of non-shiftable appliances are refrigerators, tube lights, television, etc. Shiftable-uninterruptible appliances are those appliances that can be adjusted or switched ON according to the required time slots. However, these appliances cannot be interrupted while they are functioning—for example washing machine, water heater, etc. Shiftable-interruptible appliances are those appliances that can be interrupted while they are functioning and are flexible to other time intervals. Vacuum cleaners and clothes dryers are examples of shiftable-interruptible appliances. Table 5 shows the categorization of different appliances. In addition, we have classified the appliances into five categories according to the energy usage as shown in Table 6.

For classification, we first used k-means clustering to group the users and appliances belonging to the same category. We find the closest centroid for each user and appliance to be classified and compute the mean of each cluster until the positions for the centroid do not change. Then, we train a support vector machine model on the resulting clusters and use it for classification. The algorithm for classification module is presented in Algorithm 1.
**Algorithm 1** k-means clustering and SVM for classification of users and appliancesInitialize the number of clusters *k* to assign to the input datasetInitialize *k* centroids randomly**repeat**    expectation: Assign each user and appliances to the     nearest centroid    maximize: Calculate arithmetic mean of every cluster     and assign each record to one cluster**until** centroid does not change positionTrain SVM on resulting clusterSVM for classification

#### 2.3.2. Prediction

**Figure 4:**
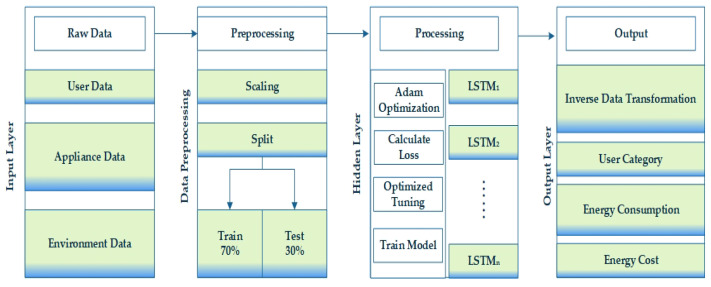
Overview of the prediction module.

In the proposed methodology, we implement the prediction module to forecast the hourly energy consumption and energy cost for each user depending on the predicted user category and the user preferences from the raw data. An overview of the prediction module is presented in Figure 4. For input, we take the user activity data, appliance usage data, and indoor and outdoor environmental condition data. We perform scaling and splitting to preprocess the data. The data are split into 70% training and 30% test set. We then use LSTM for predicting the user category of the new data and hourly energy consumption and energy cost depending on indoor environment data, outdoor weather conditions, user activity detail, and most importantly user preferences. In the processing layer, we use Adam optimization and, according to the calculated loss, we optimize the tuning of the LSTM model and train the model for forecasting the energy consumption and cost. In the output layer, we take the predicted data as the input and perform inverse data transformation and for output, we receive the user category, energy consumption, and the energy cost for each user.

**Figure 5:**
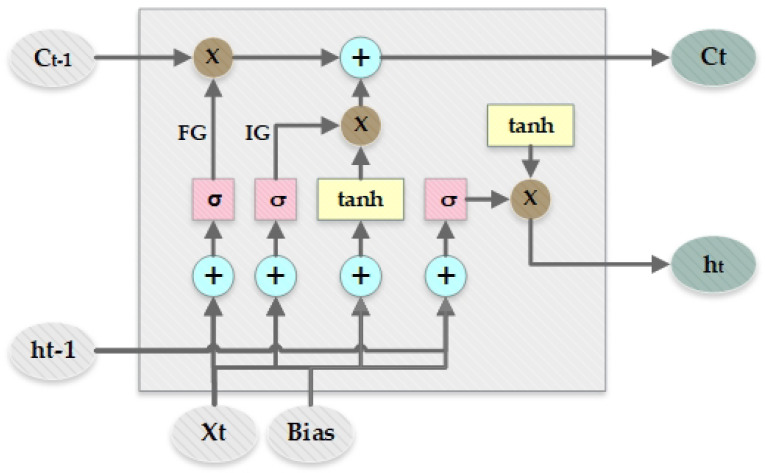
Layout of long short-term memory (LSTM).

Long short-term memory (LSTM) was introduced by Hochreiter et al. [33]. LSTM was built to avoid the problem faced by recurrent neural networks (RNN), i.e., long-term dependency problems and the problem of remembering information for a longer period of time. The layout of long short-term memory is represented in Figure 5. There are four interacting layers of neural networks in LSTM, and they are present as a chain-like structure containing repeating modules such as RNN. The core component of the LSTM is the cell state which is responsible for information flow through the whole network. Information from the cell state can be added or removed at any point in time. The forget gate (FG) or the sigmoid layer is responsible for the decision, about which information should be removed from the cell state. The forget layer takes $h_{t-1}$ and $X_{t}$ as input and outputs zeros or one for each cell state $C_{t-1}$, where zero represents the removal of information and one indicates keeping information. To determine what information is required to be stored in the cell state, the input gate (IG) or sigmoid first determines which value is needed to be updated and then a vector $C_{t}$ with new values that have to be included in the cell state is created by the tanh layer. Then, they are combined to update an existing cell state or create a new cell state.

The input gate, forget gate in the LSTM model just like any other neural network uses weights to filter or wander through information. These weights can be adjusted according to progress in learning. The formulation for LSTM network at a given time stamp *t* can be defined as in (1)

(1) $$\begin{array}{c} I_{t}=\sigma \left(M_{I}X_{t}+N_{I}h_{t-1}+P_{I}C_{t-1}+bg_{I}\right)\\ F_{t}=\sigma \left(M_{F}X_{t}+N_{F}h_{t-1}+P_{F}C_{t-1}+bg_{F}\right)\\ {\hat{C}}_{t}=\tanh\left(M_{C}X_{t}+N_{C}h_{t-1}+bg_{C}\right)\\ C_{t}=F_{t}\circ C_{t-1}+I_{t}\circ {\hat{C}}_{t}\\ O_{t}=\sigma \left(M_{O}X_{t}+N_{O}h_{t-1}+P_{O}C_{t}+bg_{O}\right)\\ h_{t}=M_{proj}^{\prime }\left(O_{t}\circ \tanh\left(C_{t}\right)\right) \end{array}$$

where σ is the sigmoid activation function; *M*, *N*, and *P* indicate the weight matrix, and bg represents the bias vectors, $M_{proj}^{\prime }$ signifies the projection matrix, and ∘ is the element-wise product.

#### 2.3.3. Optimization

For optimization, we define objective functions where our goal is to find an optimal schedule for all appliances with a maximum user preference value and minimum energy consumption and cost. Therefore, to implement an optimized schedule, we need to design an objective function that fulfills the following criteria:

- Minimizes the energy cost;
- Maximizes the higher flexibility levels (i.e., maximizes weights for Shiftable-interruptible appliances);
- Minimizes the lower flexibility level (i.e., minimizes weights for the Non-shiftable appliance class and Shiftable-uninterruptible class);
- Maximizes user preferences;
- Minimize the weight for energy consumption.

In this work, to minimize the energy cost, we also consider different seasonal and time-based tariff plans because energy pricing varies for peak hours, off-peak hours, and medium hours. It is important to focus on two goals: minimizing the basic energy cost and minimizing the energy consumption during peak hours to reduce the energy cost. For each time-based tariff, the total energy cost is calculated by applying unit price (UP) to both the basic energy rate and actual energy usage. For the basic energy rate, the current month’s basic energy rate is calculated according to the previous year’s peak load (PL). Over the current year, if PL exceeds the maximum load, then the new basic energy rate is increased over the next year. The unit price increases with the increase in usage in the case of actual energy usage for the current month. Demand charge (DC) is based on the highest level of energy the user demands at a given time. Therefore, the energy cost is calculated by multiplying PL by DC and summing it by energy consumption (EC) multiplied by the unit price as shown in (2). The objective function to minimize energy cost can be defined as in (3):

(2) EnergyCost=PL×DC+EC×UP

(3) ObjectiveFunction1:Minimize(EnergyCost).

For optimization, our second objective is to define the appliance category-based objective function. In this work, we have considered three categories for appliances: non-shiftable, shiftable-uninterruptible, and shiftable-interruptible. As non-shiftable appliances cannot be interrupted, we cannot control non-shiftable devices. Thus, our objective function focuses on the other two categories of appliances. We aim to maximize or raise the flexibility levels for shiftable-uninterruptible appliances and shiftable-interruptible appliances (i.e., maximum weights for shiftable-uninterruptible appliances and shiftable-interruptible appliances). In addition, our second aim is to minimize or lower the flexibility level of non-shiftable appliances (i.e., minimize weights for non-shiftable appliances). We consider a smart home residence with a set of *S* appliances which includes non-shiftable (NS), shiftable-interruptible (SI), and shiftable-uninterruptible (SU) appliances. Thus, total appliances are defined as in (4):

(4) S=NS+SI+SU

The entire scheduling time interval *T* of one appliance is finite, which is divided into |T| sub-intervals, e.g., 24 sub-intervals with one hour each for a day. A shiftable-uninterruptible and shiftable-interruptible appliance need to determine its working power at each sub-interval, so the consumption vector of each appliance is denoted as in (5) and (6):

(5) $$PSU_{ij}=[P_{ij}\left(1\right),P_{ij}\left(2\right),P_{ij}\left(3\right),\ldots \ldots ,P_{ij}(|T|)]\left(Shiftable-Uninterruptible\right)$$

(6) $$PSI_{ij}=[P_{ij}\left(1\right),P_{ij}\left(2\right),P_{ij}\left(3\right),\ldots \ldots ,P_{ij}(|T|)]\left(Shiftable-interruptible\right)$$

$P_{ij}\left(1\right)$ means the working power of appliance $P_{ij}$ at 1st hour. For all appliances, it can be calculated as in (7):

(7) $$PT_{ij}\left(total\right)=\sum_{k=0}^{S}\left({PSU_{ij}}^{k}+{PSI_{ij}}^{k}\right)$$

Our objective function here is to maximize appliance flexibility. For that, we first define weights for both appliance classes i.e., weight1 for shiftable-uninterruptible appliances and weight2 for Shiftable-interruptible appliances as shown in (8) and (9). Then, we maximize the appliance flexibility through (10), and the objective function is defined as in (11):

(8) $$\begin{array}{c} SU_{weights}=\alpha \left(appliance_{1}\right)+\beta \left(appliance_{2}\right)+...\\ \;\;\;\;\;\;\;\;\;\;\;\;\;\;\;\;\;\;\;\;\;\;\;\;\;\;+Y\left(appliance_{m}\right)\left(Shiftable-uninterruptible\right) \end{array}$$

(9) $$\begin{array}{c} SI_{weights}=\phi \left(appliance_{1}\right)+\phi_{1}\left(appliance_{2}\right)+...\\ \;\;\;\;\;\;\;\;\;\;\;\;\;\;\;\;\;\;\;\;\;\;\;\;\;\;+\phi_{n}\left(appliance_{n}\right)\left(Shiftable-interruptible\right) \end{array}$$

(10) $$\begin{array}{c} Maximize\left(ApplianceFlexibility\right)=\max\left(SU_{weights}\right)+\max\left(SI_{weights}\right)\\ \;\;\;\;\;\;\;\;\;\;\;\;\;\;\;\;\;ApplianceFlexibility=\Delta PT_{ij}\left(total\right) \end{array}$$

(11) $$Objective\;Function\;2:Maximize(\Delta PT_{ij}\left(total\right))$$

To give preference to user comfort, we have defined a user comfort-based objective function that focuses on a utility function to analyze user preferences and comfort. Utility function, which is a form of the sigmoid function, indicates the dissatisfaction the user experiences after an appliance usage. For example, the variation in the indoor temperature due to the heater can be defined as in (12):

(12) $$\Delta_{ij}\left(t\right)=\left|T{}_{ij}^{*}\left(t\right)-T_{ij}\left(t\right)\right|U{}_{ij}^{P}\left(t\right)=\frac{1}{1+\exp^{\theta_{ij}.\Delta_{ij}\left(t\right)-n_{ij}}}$$

where $n_{ij}$ and $\theta_{ij}$ are parameters that have been predetermined to categorize the preference of the uses. In Equation (12), $T{}_{ij}^{*}\left(t\right)-T_{ij}\left(t\right)$ indicates the actual and the desired temperature. The equation defines that, the greater the distance between the actual and desired temperature, the more discomfort the user experiences and the lesser the difference, the less the user experiences discomfort. The range of the function varies between 0 and 1.

We consider the sensor reading for humidity, temperature, $CO_{2}$, and luminosity as denoted in (13):

(13) $$\left[H_{c},T_{c},A_{c},L_{c}\right]=\left[h,t,a,l\right]$$

For each parameter, the user specifies the range of acceptable values which is considered as the user’s preferences. Ranges for user preferences are defined by user set points as in (14):

(14) $$\begin{array}{c} USP_{d}=\left(H_{d},T_{d},A_{d},L_{d}\right)\\ where,\\ H_{d}\in \left[H_{min},H_{max}\right],\\ T_{d}\in \left[T_{min},T_{max}\right],\\ A_{d}\in \left[A_{min},A_{max}\right],\\ L_{d}\in \left[L_{min},L_{max}\right] \end{array}$$

Therefore, we handle the trade-off between user preference and energy savings by defining the objective function as in (15):

(15) $$\begin{array}{c} \Delta H=|H_{max}-H_{min}|,\\ \Delta T=|T_{max}-T_{min}|,\\ \Delta A=|A_{max}-A_{min}|,\\ \Delta L=|L_{max}-L_{min}|,\\ Objective\;Function\;3:Maximize\left(\alpha_{UC}\cdot G_{UC}+\alpha_{ES}\cdot G_{ES}\right)\in \left[0,1\right] \end{array}$$

where $G_{UC}$ is a gain in user preferences and $G_{ES}$ is a gain in energy savings. $\alpha_{UC}$ and $\alpha_{ES}$ are weights for user preferences and energy savings and can be defined as fixed or adaptive so that $\alpha_{UC}+\alpha_{ES}=1$. Thus, the complete objective function to build an optimized solution can be defined as in (16)

(16) $$\begin{array}{c} Objective\;Function\;4=Objective\;Function\;1+Objective\;Function\;2+Objective\;Function\;3\\ Objective\;Function\;4=Maximize(ApplianceFlexibility,UserPreference,EnergySavings)\\ +Minimize(EnergyCost)\\ Objective\;Function\;4=Maximize(Weight1,Weight2,Weight3)+Minimize(Weight4) \end{array}$$

We have defined four weights representing part of our objective function. In the case of Weight1, all shiftable-uninterruptible and shiftable-interruptible devices are given their own weight, which is calculated in such a way that the priority of the task and user comfort is kept in mind. This equation tries to maximize the value of weight1. Weight 2 represents user preferences or comfort, Weight 3 represents levels of energy savings, and Weight 4 represents the weight for the change in energy costs. The final optimal weight is achieved by achieving weight maximization of flexible appliances, energy savings, and user comfort and minimizing the weight of electricity cost.

#### 2.3.4. Scheduling

**Figure 6:**
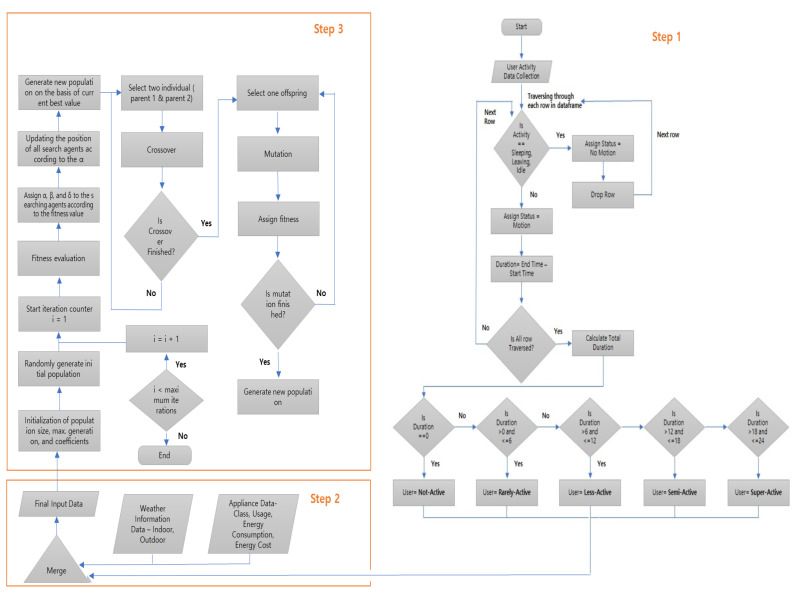
Flowchart of the scheduling algorithm.

For optimized scheduling, we have used a combination of grey wolf optimization (GWO) and particle swarm optimization (PSO). We have divided the scheduling algorithm into three steps. In step one, we obtain the classified user, i.e., super-active user, semi-active user, less-active user, rarely-active user, and not-active user. After classification, we merge the data to form the final input data. As in GWO, the first step is the initialization of population size, maximum generation, and coefficients. We then randomly generate the initial population, and, through iteration, we perform fitness evaluation. According to the fitness value, we assign α, β, and δ to the searching agents. Then, we update the position of all search agents according to α. Then, we generate a new population based on the current value. Following PSO, we performed crossover, select the offspring, perform mutation and assign fitness and, when the mutation is finished, we generate a new population or new schedule. Figure 6 shows the flowchart of the scheduling algorithm for our proposed methodology.

#### 2.3.5. Controllers

In our proposed methodology, first, we classify the user and the appliance categories for each household. Then, the system predicts the energy consumption and cost pattern for all appliances for each household for the upcoming day while giving priority to user preference. For every appliance, a predicted energy profile is generated based on the history of energy usage, user preferences, and indoor and outdoor environmental conditions. The controller aggregates these predicted energy profiles of appliances and determines the potential of each appliance in the household. The controller consists of multiple nodes connected in a tree-like structure. Every house profile is sent to the parent node which assembles all the received energy prediction profiles of each appliance. The controller then plans for each household depending on various internal and external factors and user preferences. Depending on the predicted energy consumption and cost, schedule, indoor environment condition, outdoor weather condition, and user preference, a real-time control algorithm decides the time at which appliances should be switched on or off. The controller also works around prediction errors to enhance the performance of the controller so that an optimized control is carried out that can preserve energy consumption and reduce the cost of energy.

## 3. Performance Evaluation

**Figure 7:**
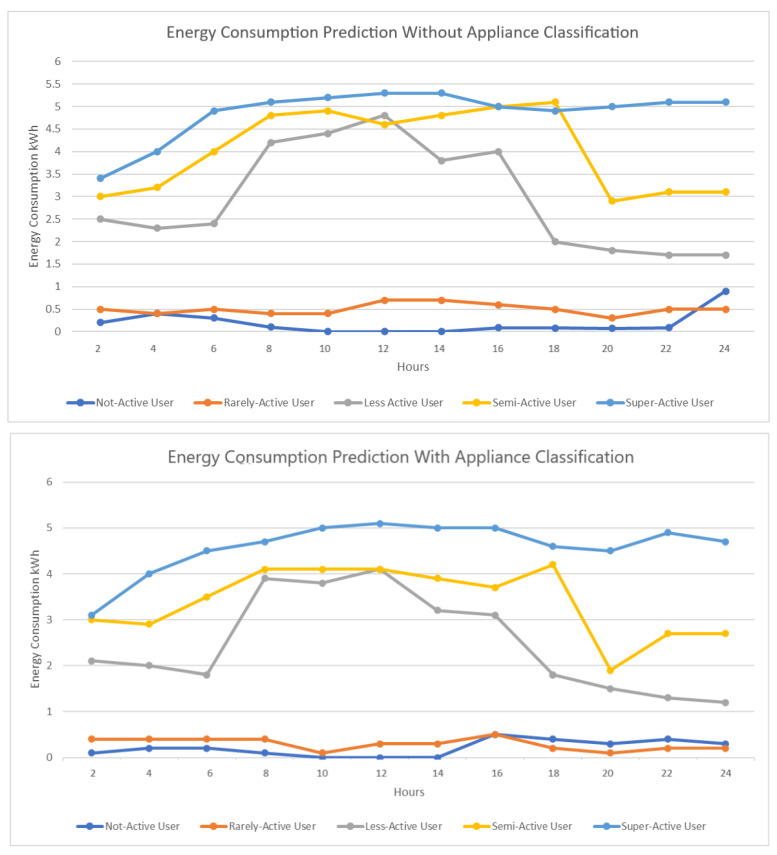
Energy consumption prediction without and with appliance classification.

In this section, we will discuss the experiments we performed to evaluate our proposed methodology and will present the results of the experiments. For showing the result, we have taken single cases of each class of user, i.e., not-active user, rarely-active user, less-active user, semi-active user, and super-active user. First, we present the energy consumption prediction results of 24 h for each user without performing any classification of appliances. Then, we predict the energy consumption of the same users with appliance classification and show how it affects energy consumption. Figure 7 presents the energy consumption prediction with and without appliance classification. As we can see from the figure, classification reduces to some extent the energy consumption for each class of user.

We then perform experiments with different models to evaluate the performance of our proposed prediction model. We find the root mean square error (RMSE), mean absolute error (MAE), and accuracy of each prediction model. For comparison, we have used GRU, RNN, GridLSTM, and CNN+LSTM.

Table 7 presents the experiment results for each prediction model.As the results depict, our proposed model performs better than other combinations of prediction models. In Table 8, we provide the comparison of prediction accuracy, MAE, and RMSE of different existing prediction methodology with our proposed approach. Results show that RF-LSTM [34] performs better than other existing models and our proposed approach improves the result to a greater extent.

**Figure 8:**
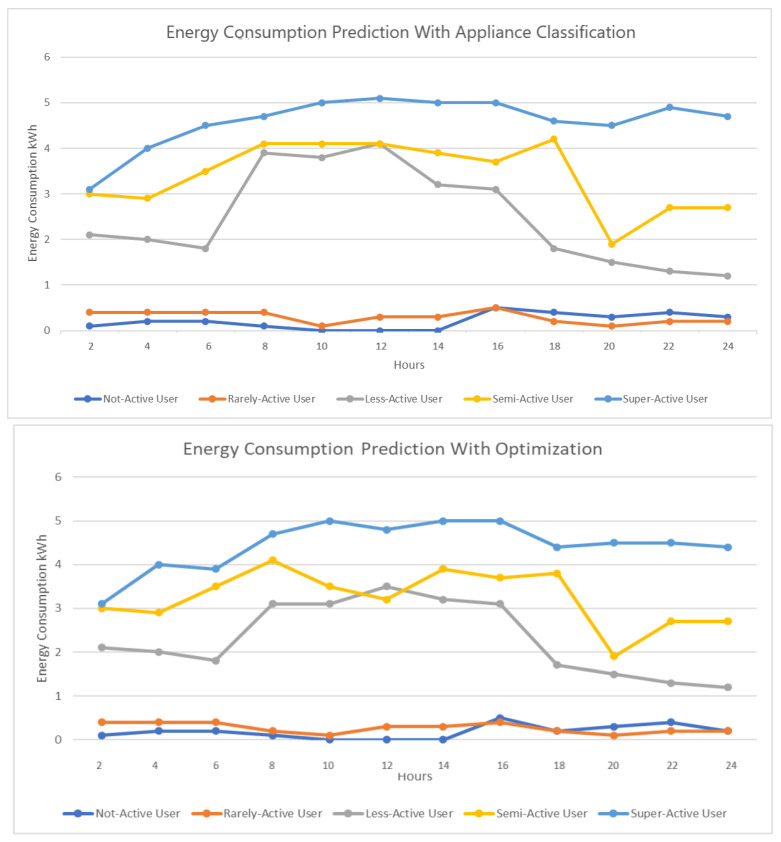
Energy consumption with optimization.

In Figure 8, we present the energy consumption with just appliance classification and energy consumption when we include optimization. As we can see for less-active, semi-active, and super-active users, there has been a reduction in energy consumption during certain hours, which indicates that optimization is a necessary step toward reducing energy consumption and therefore energy cost.

**Figure 9:**
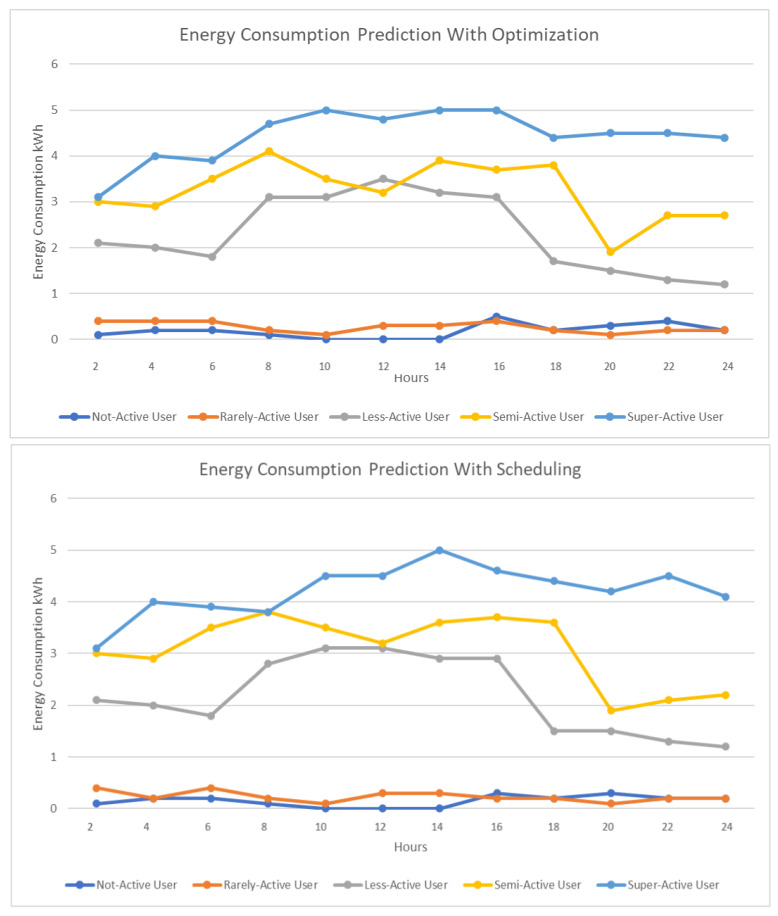
Energy consumption with scheduling.

Figure 9 represents the energy consumption when including scheduling. With a properly optimized scheduling algorithm, there is a sudden drop in the energy consumption for each user as can be seen in Figure 9.

**Figure 10:**
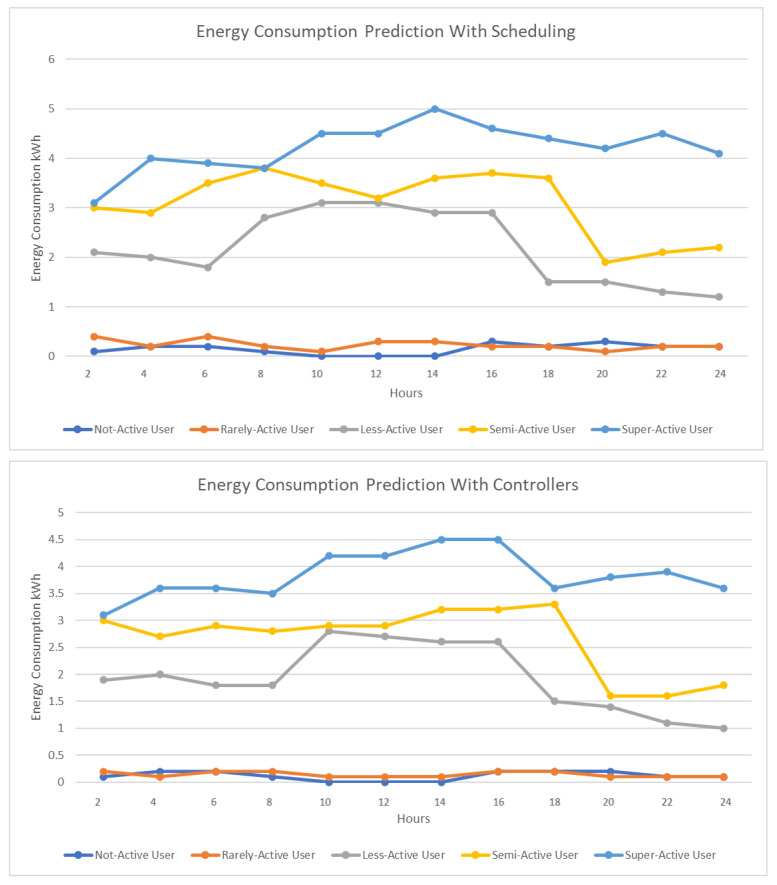
Energy consumption with controllers.

**Figure 11:**
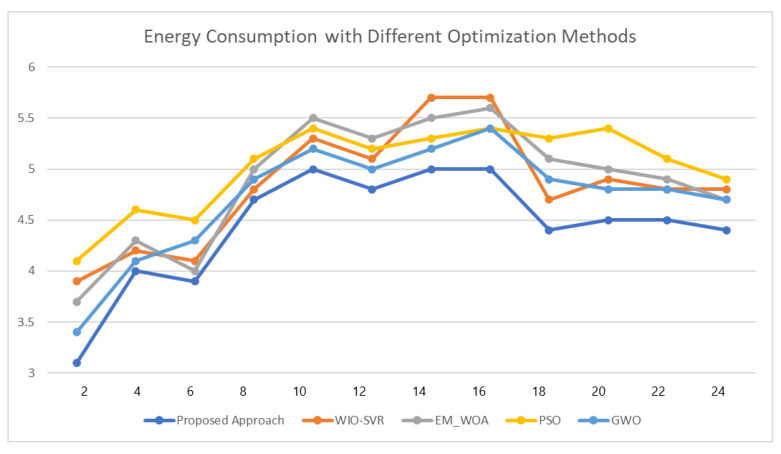
Results for energy consumption using different optimization models.

As can be seen from Figure 10, after applying classification optimization and optimized scheduling, when we use proper control rules, the energy consumption for each user reduces to a great extent and produces better results for energy consumption and cost. This indicates that our proposed model could reduce the consumption of energy and the energy cost for each category of user. In Figure 11, we use optimization models from existing studies to estimate the energy consumption and compare the result with our proposed approach. We have shown the energy consumption results for super-active user using different optimization models. The existing works that we have used for comparison are WIO-SVR [38], EM_WOA [39], PSO [40], and GWO [41].

**Figure 12:**
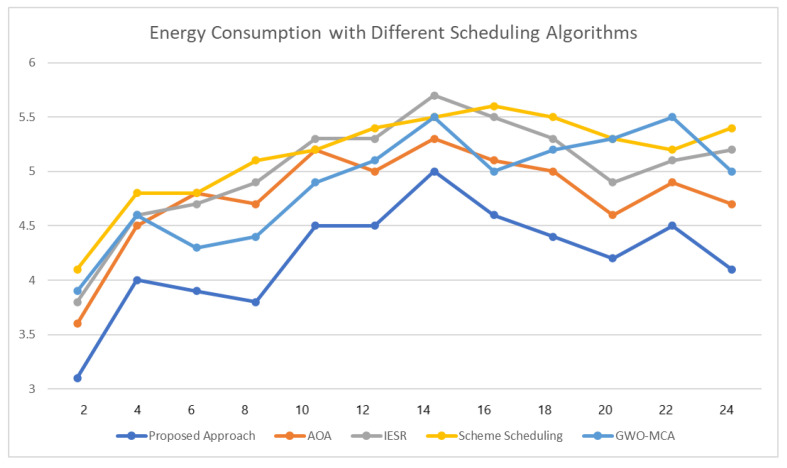
Results for energy consumption when different scheduling algorithms are used.

Figure 12 shows the comparison of energy consumption of super-active users when different scheduling algorithms are used. We have compared our proposed approach with AOA [42], IESR [43], scheme scheduling [44], and GWO-MCA [45] algorithms. As we can see from the results, our proposed approach performs better and reduces the energy consumption of users, which helps the user to reduce the cost spent for energy.

## 4. Conclusions

In this work, we have proposed five modules to find an optimal solution for smart home energy management. Our goal in this work is to reduce energy consumption and energy cost while giving priority to user preferences along with considering the types of appliances used, their consumption rates during peak hours, and indoor and outdoor environmental conditions. According to energy usage, we also classified the appliances as extremely high energy usage, high energy usage, average energy usage, low energy usage, and extremely low energy usage.

For classification, we have used k-means clustering and SVM to classify the user and appliance categories. We proposed LSTM based prediction model to predict the user category, energy consumption, and energy cost. In scheduling, we used grey wolf optimization and particle swarm optimization based algorithm for optimized scheduling of the appliances. We use control rules to control the appliances according to user preferences, weather conditions, indoor environmental conditions, and predicted scheduling outcomes. In the results section, we present the performance of our model and how each proposed module reduces the energy consumption and energy cost while considering user preferences and external factors.

Our experiments and implementation show the necessity and importance of including each module. We first present the energy consumption prediction with and without appliance classification. Results shows that classifying the appliances can reduce the consumption of energy for each user. We provided the energy consumption prediction for non-active, rarely-active, less active, semi-active and super-active users. For prediction, we have used the LSTM model and compared the accuracy, RMSE, and MAE with other models such as GridLSTM, GRU, RNN, and CNN+LSTM. LSTM performs better compared to the other models in terms of prediction accuracy and error rates. In another experiment, we present the comparison between energy consumption when we just include appliance classification and then include optimization along with classification. When we include optimization, we see a reduction in the energy consumption for less-active, semi-active, and super-active users during specific hours, which indicates the necessity of including optimization algorithms. In further experiments, we provide the results for energy consumption predictions when we include the scheduling module in our proposed methodology. For every user, we see a noticeable drop in the energy consumption and the energy cost when we include each module of the proposed methodology and apply proper control rules.

## 5. Discussion

In this work, we have proposed individual modules for optimal scheduling and control of smart home appliances. In the classification module, we divide the users into five categories so that there will be a reasonable distribution of the total number of hours on a day the user is active. For appliance classification, we have considered the amount of energy consumed by the appliance as well as the type of appliance. For the user, we categorize the user depending on the total duration of motion. For classification, we have used k-means clustering and a support vector machine. The combination of k-means clustering and SVM enhances the speed of training and classification compared to other existing models.

Since support vector uses subsets of training instances in the decision function, it is also memory efficient. In the prediction module, we use LSTM to forecast energy consumption and energy cost. We use LSTM, since, in LSTM, the complexity of updating every weight is decreased to O(1). The length of the input does not hamper the memory and time requirement. In the optimization module, we proposed novel objective functions that prioritize maximum user preferences and minimum energy consumption and cost. Our defined objective functions also take into consideration the flexibility of the appliances. For optimized scheduling, we proposed a combination of GWO and PSO which requires less parameters and also a lower number of iterations compared to other existing scheduling algorithms.

## Figures and Tables

**Table 1:** User activity sensor data feature information.

User Activity	Description
User_ID	Identification of user
Start_Time	Start time of an activity
End_Time	End time of an activity
Duration	Total duration of the activity
Location	Location of the activity (e.g., bed, cabinet, basin, toilet, shower, fridge, cupboard, toaster, microwave, seat, etc.)
Type	Type of appliance related to the activity (e.g., pressure, magnetic, electric, etc.)
Place	Place of activity in the household (e.g., bedroom, bathroom, kitchen, living, etc.)
Activity	User activity (e.g., sleeping, showering, toileting, breakfast, spare_time/TV, lunch, leaving, etc.)
Preference	User preference values for specific activity
Motion	User motion detection through sensors

**Table 2:** Sensor data feature information for the indoor environment.

Sensor	Description
Humidity	Humidity of indoor environment
Temperature	Temperature of the indoor environment
Co2	Air quality of the indoor environment
Luminosity	Light sensors for indoor environment

**Table 3:** Feature information for appliances used.

Appliance Data	Description
Appliance_ID	Identification of the appliance
Start_Time	Start time of the appliance usage
End_Time	End time of the appliance usage
Duration	Total duration of the appliance usage during the start and end time interval
Energy_Consumption	Consumption of energy by the appliance during the time interval
Energy_Cost	Cost for the consumed energy

**Table 4:** Outdoor environment feature information.

Outdoor	Description
Humidity	Humidity information for outdoor environment
Temperature	Temperature information for outdoor environment
Wind_Speed	Wind speed information for outdoor environment
Rainfall	Rainfall information for outdoor environment
Pressure	Outdoor environment pressure information

**Table 5:** Categorization of different appliances.

Appliance Category	Appliance
Non-Shiftable	Refrigerator
	Television
	Light
	Fan
	Air Conditioner
	Computer/Laptop
	Oven
Shiftable-Uninterruptible	Washing Machine
	Water Heater
	Electric Iron
Shiftable-Interruptible	Clothes Dryer
	Vacuum Cleaner
	Dishwasher

**Table 6:** Categorization of different appliances according to energy usage.

Appliance Category	Energy Usage
Extremely High Energy Usage	>10 kWh per hour
High Energy Usage	>5 kWh per hour and <= 10 kWh per hour
Average Energy Usage	>2 kWh per hour and <=5 kWh per hour
Low Energy Usage	>=1 kWh per hour and <=2 kWh per hour
Extremely Low Energy Usage	<1 kWh per hour

**Table 7:** Performance evaluation of different prediction models.

Models	RMSE	MAE	Accuracy
GridLSTM	0.83	0.76	82%
GRU	0.75	0.71	89%
RNN	0.54	0.47	93.9%
CNN+LSTM	0.49	0.37	96.2%
LSTM	0.45	0.39	98.3%

**Table 8:** Comparison of different prediction models from literature.

Models	RMSE	MAE	Accuracy
RF-LSTM [34]	0.52	0.46	91.4%
DF-DQN [35]	0.65	0.58	85.6%
Ensemble Learning [36]	0.59	0.51	88.3%
Hybrid ARIMA-GBRT [37]	0.68	0.59	83.2%
Proposed Approach	0.50	0.42	95.3%
